# Pediatric obesity and skin disease: cutaneous findings and associated quality-of-life impairments in 103 children and adolescents with obesity

**DOI:** 10.1530/EC-23-0235

**Published:** 2023-08-02

**Authors:** Laura Hasse, Dagmar Jamiolkowski, Felix Reschke, Kerstin Kapitzke, Jantje Weiskorn, Olga Kordonouri, Torben Biester, Hagen Ott

**Affiliations:** 1 Department of Pediatric Dermatology and Allergology, Children’s Hospital Auf der Bult, Hannover, Germany; 2 Department of Pediatric Diabetology, Children’s Hospital Auf der Bult, Hannover, Germany

**Keywords:** acanthosis nigricans, keratosis pilaris, striae distensae, quality of life, BMI

## Abstract

**Objective:**

Little is known about specific cutaneous findings in children and adolescents with overweight and obesity. This study assessed the association of skin signs with pivotal auxological and endocrinological parameters and their influence on the quality of life (QoL) of young people with obesity.

**Study design:**

All patients initially recruited for a tertiary hospital's weight control program were offered participation in this interdisciplinary, single-center, cross-sectional study. All participants underwent a detailed dermatological examination, anthropometric measurements and laboratory examinations. QoL was assessed with validated questionnaires.

**Results:**

A total of 103 children and adolescents (age 11.6 ±2.5 years, 41% female, 25% prepubertal, BMI SDS 2.6 ± 0.5, homeostatic model assessment (HOMA) score 3.3 ± 4.2; mean ± s.d.) were recruited in a 12-month study period. Skin affections were linearly associated with increasing BMI and higher age. The most common skin findings were (%) striae distensae (71.0), keratosis pilaris (64.7), acanthosis nigricans (45.0), acne vulgaris (39.2), acrochordons (25.5) and plantar hyperkeratosis (17.6). The HOMA score was associated with acanthosis nigricans (*P = *0.047), keratosis pilaris (*P = *0.019) and acne vulgaris (*P <* 0.001). The general mean QoL(QoL) score, as assessed by the WHO-5, was 70 out of 100. A total of 38.9% of participants reported impaired dermatological QoL.

**Conclusions:**

This study shows the high prevalence of skin lesions in children and adolescents with obesity. The association between skin lesions and the HOMA score indicates that skin manifestations are a marker of insulin resistance. To prevent secondary diseases and improve QoL, thorough skin examinations and interdisciplinary cooperation are necessary.

## Background

The prevalence of obesity is steadily increasing in children and adolescents and has become a global health burden in recent decades. In Germany, the current prevalence of overweight among minors is 15.4% and that of obesity is 5.9%, without sex differences (1). This trend has increased significantly during the coronavirus-19 pandemic, and the prevalence of diabetes has also increased continuously in recent years (2). According to the World Health Organization (WHO), the global prevalence of overweight and obesity among children aged 5–19 years is approximately 18% (https://www.who.int/news-room/fact-sheets/detail/obesity-and-overweight). In contrast to adulthood obesity, which is defined as a fixed value of 30 kg/m² body weight and above, childhood obesity is characterized as a body mass index (BMI) over the 97th sex- and age-related reference percentile (3).

In individuals with childhood obesity, health-related quality of life (QoL) is impaired (4, 5) and pivotal hormone and metabolic pathways are negatively influenced. As a consequence, children and adolescents with obesity are at high risk of concomitant and secondary dysfunctions in several organ systems, resulting in high blood pressure, diabetes, fatty liver, orthopedic disorders, and psychological burden (6, 7). In addition, the skin represents an important target organ, and several papers have described dermatological pathologies in adults with overweight and obesity (8, 9, 10). While some specific entities, such as acanthosis nigricans (AN), have been studied in individuals with childhood obesity (11, 12), structured investigations of skin abnormalities in pediatric patients with obesity are still scarce. Moreover, the impact of cutaneous afflictions on the general well-being and skin-related QoL of children with obesity has not been extensively studied thus far (13, 14, 15, 16). Similarly, associations of skin diseases with anthropometric data and metabolic parameters have not yet been described. Therefore, the aim of this study was to:

- describe the prevalence and clinical characteristics of skin disorders in children and adolescents with obesity,
- evaluate the impact of these skin disorders on the QoL of the included subjects, and
- establish possible associations of skin disorders with anthropometric and metabolic data.

## Study design and participants

The study was planned as a prospective, monocentric and observational cross-sectional study of a defined cohort without intervention. The study was conducted under the ethical principles of the Declaration of Helsinki and was approved by the Ethical Committee at Hanover Medical School (reference number: 9528_BO_S_2020).

### Study setting

In a tertiary care children’s hospital, pediatric patients with obesity had the opportunity to participate in a 1-year multidisciplinary training program for weight control (17). As part of the initial recruitment examination for this program, all patients (aged 1–18 years) were offered an additional dermatological examination. Study participation was proposed to each patient once during our 12-month study period (January–December 2021). Patients were included as study participants after receiving detailed verbal and written information, and after they, along with at least one parent or guardian, had signed the consent form. Exclusion criteria were previous participation in any other weight loss program and the manifestation of endocrinological disorders, including hyperandrogenism, except diabetes.

### Interdisciplinary patient evaluation

Upon inclusion, the following anthropometric data were recorded: body weight and height, BMI, waist circumference, skinfold thickness of the scapula and triceps, blood pressure and Tanner pubertal stage. Hypertension was assessed using sex- and percentile-specific reference values (18). The standardized BMI (BMI SDS) was used to more accurately reflect overweight by illustrating the deviation of the BMI above the age- and sex-specific median BMI value. To obtain more precise information on body composition, a bioimpedance analysis (BIA) was also performed (Nutribox, Data Input GmbH, Pöcking) (19). Thus, body fat (BF), total body water (TBW), lean body mass (LBM) and phase angles were assessed as measurement parameters for cell function (20).

Blood withdrawal was performed after overnight fasting to allow for the analysis of the following parameters: low-density protein cholesterol (LDL-C), high-density protein cholesterol (HDL-C), total cholesterol (TC), triglycerides, γ-GT, GOT, GPT, blood urea nitrogen, creatinine, uric acid, TSH, HbA1c, fasting glucose and fasting plasma insulin. Insulin resistance (IR) was calculated using the homeostatic model assessment (HOMA) according to the original formula of Matthews *et al.* (21) and defined by a HOMA score above 2.5. Data on preexisting diseases and allergies were obtained from medical records.

Each participant underwent a standardized dermatological examination of the entire integumentary system, including the skin, scalp and oral mucosa, by the dermatology clinic team. All skin lesions were documented regarding their morphology and location and were recorded photographically.

QoL was assessed with the following validated questionnaires:

The WHO-5 Well-being Index, designed by the WHO, captures social, mental and physical well-being (22). The final score is between 0 and 100, ranging from the worst imaginable well-being to the best imaginable well-being.

The dermatological quality of life (dQol) index was assessed with the Children's Dermatology Life Quality Index (CDLQI), which has been used in numerous studies to measure skin disease symptoms and therapy in the 7 days prior to filling out the questionnaire (23, 24). The self-explanatory questionnaire consists of tenquestions covering symptoms, feelings and influences on daily life, such as friendships, clothing, recreational activities, sports, school and therapy. Each question can be answered with the following options: 3 (‘very much’), 2 (‘quite a lot’), 1 (‘only a little’) and 0 (‘not at all’). The final score is between 0 and 30 points, ranging from no impairment to a very severe impairment of QoL due to the skin condition.

Furthermore, the participants completed the Self-Perception Profile for Children (SPPC) (25). The modified SPPC includes five different subscales (scholastic competence, social competence, physical appearance, behavioral conduct and global self-worth) with six different items. Each item can be rated from ‘very true for me’, ‘sort of true’, ‘not very true’ to ‘not at all true’. The items are scored from 1 to 4, whereby 4 represents the most adequate self-judgment and 1 represents the least adequate self-judgment. By taking a separate look at each subscale, we were able to analyze the specific self-concept domains individually and their contribution to global self-worth, which was scored separately.

### Statistics

All data collected in the study were pseudonymized. A descriptive statistical analysis was performed to assess the prevalence of each skin abnormality. All analyses were performed using the statistical software Statistical Package for the Social Sciences (SPSS) Version 28.0.1.1.

Descriptive results are presented as the mean and standard deviation (s.d.) for normally distributed parameters or the median and range for nonnormally distributed parameters, absolute numbers or valid percentages. To investigate the influence of the anthropometric and laboratory parameters on skin changes, Pearson’s correlation test and linear regression were applied. *P* < 0.05 was considered statistically significant.

## Results

### Study population

A total of 134 patients were screened from January to December 2021, of whom 31 were excluded due to previous weight reduction training or a concurrent endocrinological disease other than diabetes. As a result, 103 participants, including 61 boys (59%) and 42 girls (41%), were recruited for the study. Of these participants, 25% were prepubertal. With a mean waist circumference of 100 cm, a mean subscapular skinfold thickness of 3.1 cm and a mean triceps skinfold thickness of 3.2 cm, the study population was considerably overweight. The BIA revealed a mean BF of 26.84 kg (35.36%), TBW of 38.43 L and LBM of 47.8 kg. Arterial hypertension was found in approximately half of the study population (47.5%). Detailed demographic and anthropometric data are provided in Table 1.

**Table 1:** Characteristics of the study population.

	*n* (%)	Age mean (s.d.)	Female* n* (%)	BMI SDSmean (S.D.)	BF % mean (S.D.)	Hypertension *n* (%)
Whole study population	103 (100)	11.6 (2.5)	42 (40.8)	2.6 (0.5)	35.4 (4.5)	49 (47.5)
<10 years	24 (23)	8.5 (1.4)	12 (50)	2.8 (0.5)	35.0 (5.0)	13 (52)
10–14 years	59 (57)	11.6 (1.1)	20 (34)	2.5 (0.5)	35.2 (4.1)	26 (44)
>14–17 years	20 (19)	15.4 (0.9)	10 (50)	2.8 (0.5)	36.0 (5.3)	10 (50)

BF, body fat.

The median HOMA score was 3.3, and 52 (55.3%) participants showed IR. Six (5.9%) participants had preexisting type 2 diabetes mellitus, and one (1%) was diagnosed at this time. Seventeen (16.7%) subjects had hypercholesterolemia, 13 (12.9%) subjects had dyslipidemia, and 4 (3.9%) subjects had hyperlipidemia. In total, 17 (16.7%) were found to have nonalcoholic fatty liver disease (NAFLD). The results of further laboratory tests are shown in Table 2.

**Table 2:** Laboratory results presented as median (interquartile range).

Parameter	Whole study population	<10 years	10–14 years	>14 years
LDL (mg/dL)	96.5 (18–190)	105 (49–183)	97 (18–190)	95 (66–151)
HDL (mg/dL)	49 (30–111)	50 (36–79)	52 (30–111)	48 (33–77)
Total cholesterol (mg/dL)	169 (81–265)	174 (112–265)	169 (81–265)	164 (115–216)
Triglycerides (mg/dL)	79.5 (17–379)	88 (29–200)	99 (17–379)	104 (79–241)
GOT (U/L)	26 (10–118)	28 (11–43)	29 (11–118)	22 (10–40)
GPT (U/L)	38.5 (16–250)	40 (20–98)	50 (16–250)	39 (20–65)
GGT (U/L)	18 (6–58)	17 (6–39)	20 (10–58)	22 (10–48)
Fasting glucose (mg/dL)	88 (71–318)	85 (71–101)	92 (78–264)	197 (76–318)
Urea (mg/dL)	25 (11–46)	25 (11–36)	26 (13–46)	26 (15–43)
Creatinine (mg/dL)	0.5 (0.3–1.0)	0.4 (0.3–0.5)	0.6 (0.3–0.9)	0.6 (0.4–1.0)
Uric acid (mg/dL)	5.0 (1.9–8.8)	4.3 (2.2–5.9)	5 (1.0–8.8)	5.5. (2.9–8.3)
TSH (µU/mL)	2.3 (0.8–8.0)	2.2 (0.8–4.4)	2.5 (0.9–8.0)	0.8 (4.8–2.0)
HbA1c (%)	5.5 (4.1–11.7)	5.4 (4.5–5.9)	5.5. (4.1–7.0)	4.9 (4.9–11.7)
Fasting insulin (µU/mL)	15.0 (1.0–85.6)	16.8 (1.0–85.6)	21.1 (2.8–81.5)	23.1 (2.7–85.2)
HOMA	4.6 (0.0–14.2)	3.6 (0–21.3)	4.8 (0.6–19.5)	5.5. (21.9–24.2)

Physical examination revealed hyperlordosis of the lumbar spine in 33 (32.4%) participants, kyphosis of the thoracic spine in 48 (47.5%) participants and scoliosis in 3 (3%) participants. Furthermore, 18 (17.8%) patients had flat feet, of whom 11 (11%) also showed fallen arches. Twenty-five individuals (24.8%) presented with valgus knees.

Other extracutaneous comorbidities were observed in a small number of the recruited participants: lipomastia (*n = *3, 3%), chronic bronchitis (6, 5.8%), allergic rhinoconjunctivitis (2, 1.9%) and other allergic diseases (2, 1.9%). None of the study participants reported smoking or the use of hormonal contraception.

### Skin changes

**Figure 1:**
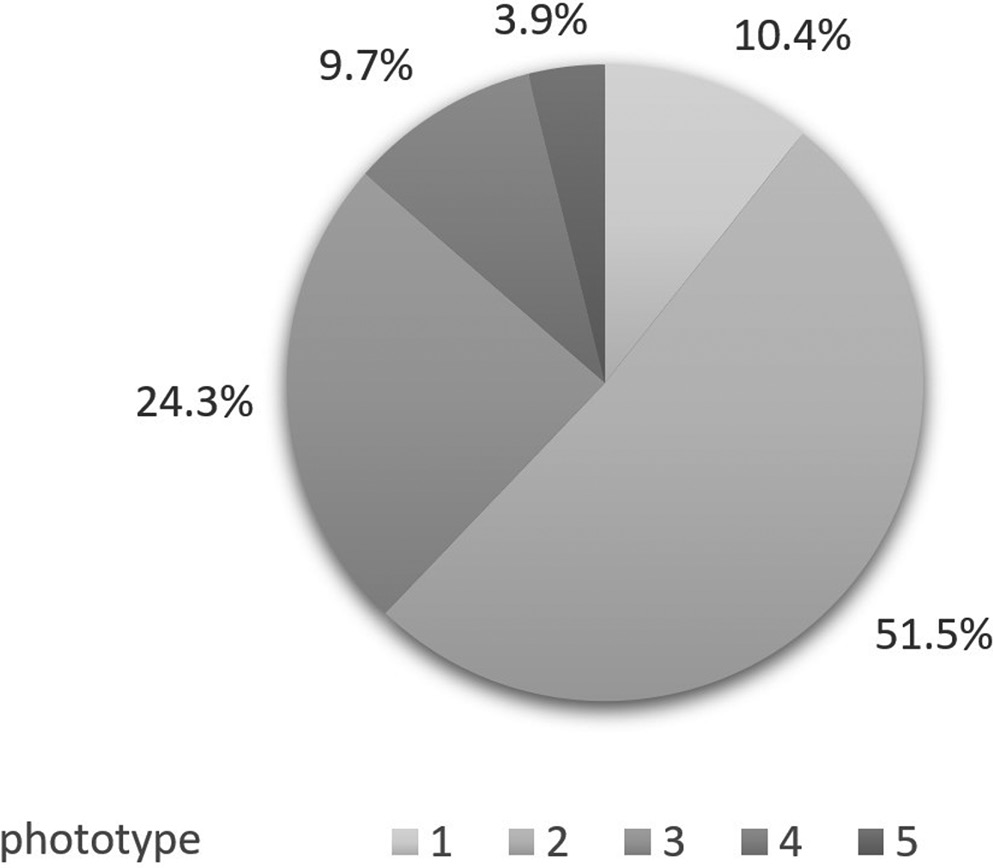
Distribution of skin phototypes in the study population according to the Fitzpatrick scale.

Of all participants, 88% had skin phototypes 1–3, which represent the most common phototypes in German minors, whereas only 9% and 4% had phototype 4 or 5, respectively (Fig. 1). Upon inclusion, 13 participants (12.6%) were undergoing dermatological treatment for atopic dermatitis (*n = *4, 3.8%) and other skin diseases, e.g., psoriasis (*n = *1, 1.0%) or suspected hair loss (*n = *1, 1.0%).

**Figure 2:**
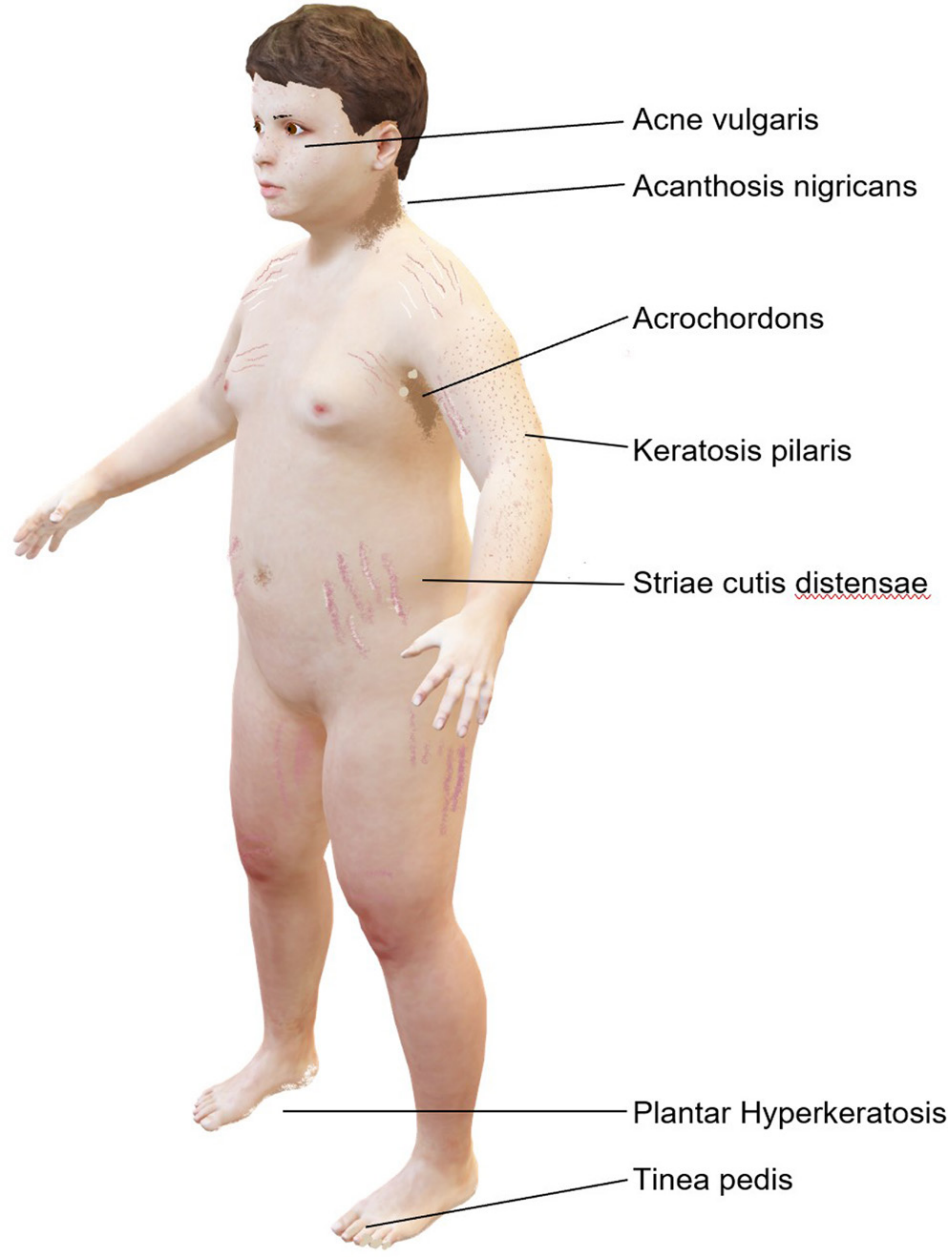
Schematic representation of typical skin changes in children and adolescents with overweight and obesity*. *For a clear representation of skin changes, only skin type 1 is illustrated. Skin changes can occur in all skin types. External sex characteristics are not shown. All sex entities should be explicitly addressed.

**Figure 3:**
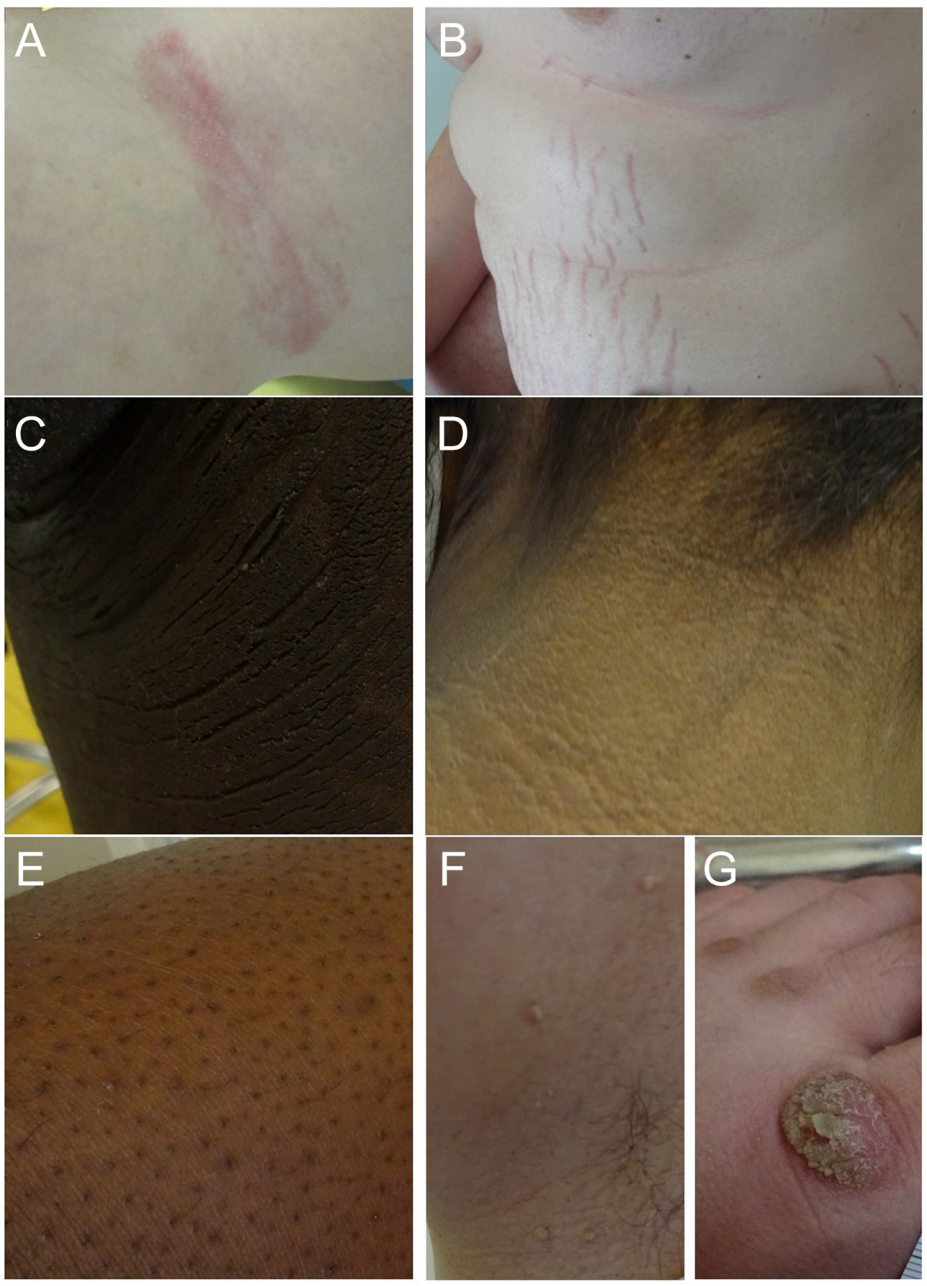
Selection of skin changes. (A) Intertrigo; (B) abdominal striae cutis distensae; (C) axillary acanthosis nigricans; (D) nuchal acanthosis nigricans; (E) keratosis pilaris; (F) axillary acrochordons; (G) hyperkeratosis due to tight footwear.

A total of 102 (99%) subjects revealed skin changes (Figs 2 and 3) that are presented in more detail in Table 3. Generally, the prevalence of skin changes significantly increased with age (*r = *0.467, *P <* 0.001) and BMI SDS (*r = *0.426, *P <* 0.001).

**Table 3:** Complete spectrum of skin disorders detected in 103 pediatric patients with obesity.

Skin disorder	*n* (%)
Xerosis cutis	68 (66.0)
Striae distensae Abdomen Upper arm Back Mammae Thigh Hips Others (axilla, shoulder, knee)	65 (63.1) 52 (50.5) 35 (33.9) 26 (25.2) 19 (18.4) 12 (11.6) 5 (4.9) 43 (41.7)
Kerarosis pilartis	65 (63.1)
Acanthosis nigricans	46 (44.7)
Acne vulgaris	42 (40.8)
Scalp desquamation	28 (27.2)
Acrochordons	26 (25.2)
Café-au lait spot	22 (21.4)
PH	18 (17.5)
Naevi	16 (15.5)
Eczema	15 (14.6)
Tinea pedis	12 (11.7)
Atrophic scars	12 (11.7)
Verrucae vulgares	11 (10.7)
Folliculitis	11 (10.7)
Intertrigo	11 (10.7)
Telangiectasia	9 (8.7)
Hirsutism	6 (5.8)
Pityriasis versicolor	4 (3.9)
Atopic dermatitis	4 (3.9)
Seborrheic dermatitis	2 (1.9)
Digital hyperkeratosis	2 (1.9)
Cellulitis	2 (1.9)
Naevus depigmentosus	2 (1.9)

#### Association of skin disorders with anthropometric and laboratory parameters

In total, 68 (66%) subjects had dry skin (xerosis cutis), while 65 (63.1%) individuals presented with keratosis pilaris (KP), which occurred more frequently in subjects with increased HOMA scores (*r = *0.210, *P = *0.019) and correlated with the BMI SDS (*r = *0.825, *P = *0.03).

Pruritus was described by 46 (44.7%) participants, of whom 44 subjects reported mild itching. Moderate and severe pruritus was recorded in 1 patient. Pruritus occurred most frequently in association with xerosis cutis (68%) and KP (68%), although the correlation of these entities was not statistically significant. Moderate pruritus was reported by one subject who had KP, and severe pruritus was reported by one subject with marked xerosis cutis. Of the participants with preexisting atopic dermatitis, only 9 out of 15 (60%) suffered from pruritus.

Acne vulgaris (AV) was present in 42 (40.8%) patients, of whom 3 (2.9%) had a severe phenotype (acne conglobata). Acne prevalence significantly correlated with the HOMA index (*r = *0.354, *P <* 0.001), fasting insulin (*r = *0.375, *P <* 0.001) and triglyceride values (*r = *0.266, *P = *0.004). Likewise, a positive correlation of skin disorders with patient age (*r = *0.480, *P <* 0.001) and IR (*P = *0.009), but not with the BMI SDS, was found.

Acrochordons were present in 26 children and adolescents (25.2%), preferentially occurred in the axillary regions and varied in number (from 1 to 7 lesions per patient). No correlations with anthropometric or laboratory parameters were observed. Plantar hyperkeratosis was seen in 18 (17.5%) subjects, and 2 (1.9%) children showed hyperkeratosis of the toes due to tight footwear.

The most common skin infections in the studied population were tinea pedis (11.7%), followed by verrucae vulgares and folliculitis (10.7%). Folliculitis was observed more frequently in participants with a HOMA score within the IR range (*r = *0.175, *P = *0.046).

After linear regression between the degree of obesity, as defined by the BMI SDS, and skin changes, a significant correlation was found for striae distensae (*r = *0.334, *P <* 0.001), AN (*r = *0.195, *P = *0.049) and hypertrichosis (*r = *0.240, *P = *0.015).

Striae distensae were observed in 65 subjects (63%) and occurred primarily in the abdominal region, followed by the brachial and dorsal regions. A significant correlation was found between striae and the results of the BIA (*r = *0.354, *P <* 0.001), i.e. increased proportions of BF, TBW and LBM. Additionally, striae were significantly more common in patients with a higher BMI SDS (*r = *0.334, *P <* 0.001) and thicker skin folds in the triceps (*r = *0.302, *P = *0.005) or scapula (*r = *0.377, *P <* 0.001) regions.

In total, 46 participants (44.7%) had AN. Of these participants, two-thirds (62,9%) had skin phototype 3 or higher (2 individuals (4.2%) with skin phototype (SPT) 1, 15 individuals (32.6%) with SPT 2, 16 individuals (34.7%) with SPT 3, 9 individuals (19.6%) with SPT 4 and 4 individuals (8.6%) with SPT 5). The skin types of the participants without AN were strikingly lighter. In total, 9 individuals (15.8%) had skin type 1, 38 individuals (66.8%) had skin type 2, 9 individuals (15.8%) had skin type 3, and 1 individual (1.7%) had skin type 4. Additionally, the HOMA index score (*r = *0.174, *P = *0.047) and BMI SDS (*r = * 0.195, *P = *0.049) were significantly higher in subjects with AN.

### General and skin-related quality of life

#### General well-being

Most patients had unimpaired or largely unimpaired general well-being as determined by a mean WHO-5 questionnaire score of 70/100 points or higher as well as by mean scores of 19–25 points and 13–18 points in 44 (45.9%) and 40 (41.7%) participants, respectively. Moderately reduced well-being (10–12 points) was reported by 6 (6.2%) individuals, while markedly limited (<10 points) and very limited well-being (<7 points) was reported by 3 (3.1%) subjects.

The mean global self-worth and behavioral conduct scores, assessed by the SPPC, were slightly lower in our study group than in children of the same age in the general population (25^26^). Of the specific SPPC domains, self-perceived physical appearance showed the greatest reduction, with a mean score of 2.13. Likewise, both the behavioral conduct domain (2.76) and the general perception of global self-worth scores were reduced (2.73) in our study group compared to children of the same age.

#### Skin-related self-perception and QoL

While general well-being and global self-worth were not associated with the number of skin changes, a correlation between skin lesions and the score on the SPPC physical appearance subdomain was found (*r = *−0.278, *P = *0.007). As measured with the CDLQI score, 62 patients (60.1%) did not show an effect of cutaneous lesions on their QoL (0–1 points), whereas 32 (31.1%) individuals experienced a small effect (2–6 points), and another 8 (7.8%) participants showed moderate QoL reductions (7–12 points). Only one participant with severe AN displayed a very strong QoL impairment (16 points), while no individual experienced an extremely large effect (CDLQI score of 19–30 points). As a result, the mean CDLQI score of the entire study group was 2 (range 0–16), without statistically significant sex differences. A positive correlation could be established between the CDLQI score and the following cutaneous affections: pruritus (*r = *0.437, *P <* 0.001), striae (*r = *0.220, *P = *0.013), hirsutism (*r = *0.399, *P <* 0.001), dermatitis (*r = *0.250, *P = *0.005), acne conglobate (*r = *0.177, *P = *0.037) and folliculitis (0.167, *P = *0.045). Generally, a higher number of skin changes was associated with a more pronounced QoL impairment (*r = *0.273, *P = *0.03).

## Discussion

To the best of our knowledge, this is the largest study of the clinical spectrum of cutaneous lesions, their associations with anthropometric data and their impacts on QoL in children and adolescents with obesity. Compared to a recent study including 82 pediatric patients with overweight and obesity, the mean BMI SDS values were similar (2.74 ± 0.72 vs 2.60 ± 0.5) (15). Furthermore, four studies with a similar dermatological focus and >80 included patients (*n =* 82 (14), *n = *100 (26), *n = *510 (27), *n = *91 (16)) did not report the BMI SDS values of the recruited patients.

### Skin conditions associated with obesity

Striae represented the most common skin condition in the present cohort and were significantly more frequent in patients with a higher BMI SDS and increased proportion of BF. This prevalence was comparable to that in all but one of the abovementioned studies. Gupta and coworkers reported a markedly lower striae prevalence (21.1%), which is most likely due to milder obesity in nearly two-thirds of the reported population (26). Interestingly, our phase angle measurements did not provide any evidence of reduced tissue quality or impaired cell membrane function, which further underlines that striae may not be linked to secondary tissue damage but rather to rapid weight gain and skin expansion in individuals with obesity (9, 28).

Plantar hyperkeratosis (PH) is rare in otherwise healthy children and adolescents without obesity but was encountered in every sixth patient in the present study. In adults with overweight and obesity, PH occurs more often due to prolonged weight bearing over a period of years (10). PH frequency is known to depend on the grade of obesity (29), which explains the varying prevalence (3.7% - 45.1%) in heterogeneous study populations (15, 26, 27, 30). Of note, orthopedic abnormalities did not influence plantar hyperkeratosis, as valgus knees and foot malpositioning occurred independently of plantar hyperkeratosis in our study population.

Certain skin infections are known to occur more frequently in individuals with obesity (31). Accordingly, the prevalence of tinea pedis (12%) is markedly elevated in children with obesity compared to schoolchildren in the general population, in whom the prevalence ranges from 2.69% (32) to 5.7% (33). While dermatomycosis occurred substantially more frequently in Egyptian children (21.9%), the results of other previous studies are comparable to the general population (15, 16). Similarly, in another German study examining minors with diabetes mellitus, tinea pedis was observed only in 1.6% of the participants (34). It has been estimated that 30% of all children and adolescents will develop cutaneous warts before adulthood (35), which have been detected in up to 44% of preschool children (36). In contrast, the prevalence of warts was low in the present study population (10.7%). The prevalence of folliculitis in this study was similar to that in a recent Canadian investigation (16) and markedly lower than that in a Turkish study in children with similar BMI SDS values (15). In addition to ethnic influences, climatic influences or differences in health care systems, e.g., access to topical antimicrobial therapy or dermatological care, may account for this difference in folliculitis prevalence. Nevertheless, these data underline the strongly elevated prevalence of folliculitis in young patients with obesity compared to the estimated prevalence in the general population (14).

In individuals with obesity, intertrigo is due to unusually deep skin folds facilitating transpiration, skin irritation and fungal infections (15, 31). We detected a prevalence of intertrigo of 10.7%, which is in line with previous investigations (15, 37) but has not been investigated in other similar studies (14, 16).

### Skin and insulin resistance

Obesity and IR are closely linked, and the skin is a known target organ of IR and hyperinsulinemia. Accordingly, elevated insulin-like growth factor (IGF) levels lead to the proliferation of keratinocytes and fibroblasts and thus promote AN (38). AN is therefore considered an important cutaneous marker of IR not only in adulthood but also in childhood and adolescence (39, 40). We were able to corroborate previous findings (40, 41) by demonstrating a significant correlation between the HOMA-IR value and the occurrence of AN. While the prevalence of AN in our study was comparable to that found in Egyptian children (27), it was higher in studies of Turkish (63.4%) (15) and Canadian (68%) (16) children with obesity. Whether this discrepancy was due to different HOMA-IR results could not be analyzed because this parameter was not reported in the abovementioned studies (15, 16). In line with previous studies, we detected AN more frequently in children with higher skin phototypes in whom the HOMA index score was also elevated compared to individuals with lower skin phototypes (42, 43).

KP was twice as common in this study as in other studies, in which the prevalence ranges from 8.7% to 42% (15, 16, 27). This cornification disorder is associated with high BMI (8, 44) and is linked to IR in young adults (8, 45). Additionally, our results confirm the association between KP and both BMI SDS and increased HOMA-IR values in children and adolescents with obesity. In line with Yosipovitch *et al.* (8), we observed that KP was significantly associated with dry skin. The prevalence of xerosis cutis in the mentioned studies was also substantially lower (15, 16, 27), also explaining the lower prevalence of KP. Differences in the prevalence may also occur due to genetic variations (44), and KP may improve through sun exposure.

Acne represents a multifactorial skin disease with a variety of influencing factors, such as hormonal changes, nutrition and genetic predisposition (46). As only 25% of the study population was prepubertal, the observed acne prevalence was very similar to that in the general population (47). Interestingly, the correlations with HOMA, fasting insulin and triglyceride values give indications of the relationship between the metabolic profile and acne occurrence (46). Compatible with findings in the literature (48), the results of this study revealed clear correlations between the development of acne and the occurrence of IR. This can be explained by the increased levels of IGF in individuals with IR and describes the dependence on IGF in the development of acne (49).

### General well-being and quality of life

To date, this study is the first to investigate general well-being and disease-related QoL in children and adolescents with obesity. Only one study with a smaller cohort (*n = *90) examining dQoL in minors with overweight and obesity has been published thus far (16). The authors describe a median CDLQI score of 2, which is slightly poorer than that in our study population (median: 1). Nevertheless, the cited study did not specify dQoL in individuals with specific skin lesions and did not address possible reasons for QoL impairment.

In the current study, the impact of cutaneous lesions on the patients’ QoL was low, as QoL was unaffected (60.1%) or barely affected (31.1%) in a large proportion of participants. However, 8.8% of the participants suffered from moderate-to-severe dQoL impairment. Regarding specific skin changes, patients with xerosis cutis or pruritus were particularly significantly affected by dQoL reductions. In particular, skin changes causing itching or pain and clearly visible lesions affecting the external appearance (e.g. striae) were associated with impaired dQoL. This is in accordance with other studies on cutaneous itching (50, 51) and psychological impairment due to striae (52). Compared to a study examining skin lesions in children and adolescents with diabetes (34), we observed that CDLQI scores were slightly poorer in our study population. In the comparison of the skin conditions of both study populations, it was obvious that the severity of skin changes was higher in our study population, explaining the more severe influence on QoL. Most of the study population revealed no or only a very limited reduction in general well-being (91%). However, self-perceived physical appearance was significantly impaired in individuals with a higher burden of skin lesions. These findings suggest that impaired QoL in children with obesity may be related to disturbed self-confidence due to clearly visible and/or pruritic skin lesions.

### Study limitations and strengths

As this investigation was performed in a specialized outpatient clinic, we did not include a control group of individuals with normal body weight. Second, we did not document the socioeconomic status or ethnic background of the participants and their parents, which could influence cutaneous lesions in terms of differences in hygiene and skin care routines. Likewise, differences in education and parental income levels, which potentially influence diet and knowledge of health-promoting behaviors, were not recorded.

As a major strength, we present data generated in an interdisciplinary setting of pediatric endocrinologists and dermatologists accounting for a comprehensive assessment of skin lesions. Moreover, outpatient attendance over an entire year was investigated, and QoL parameters, as well as anthropomorphic data, metabolic parameters and BIA results were reported, which is, to the best of our knowledge, new in this field.

## Conclusion

This is the first interdisciplinary study of skin changes in children and adolescents with overweight and obesity involving pediatricians as well as dermatologists and endocrinologists. In this investigation, data on anthropometric and metabolic parameters were simultaneously recorded along with the general and dQoL parameters, which were comparable to the results from our colleagues Albaghdadi *et al.* (16); in addition, our study captured general well-being. As the first Western European study on this topic, it shows differences in the frequency of skin lesions and establishes correlations with metabolic data. Skin changes such as acanthosis nigricans or tinea pedis must be considered early predictors of secondary diseases caused by obesity. To reduce preventable concomitant disease and the impact of skin changes on QoL, a full body skin inspection, especially focusing on predilection sites such as intertriginous and interdigital areas, is of pivotal importance in children with overweight and obesity. If a detailed skin examination reveals lesions that are potentially associated with secondary obesity-related diseases, especially acanthosis nigricans, additional diagnostic tests are mandatory (e.g. insulin serum levels, genetic testing) (53).

## Declaration of interest

All authors declare no COI in association with this manuscript.

## Funding

There was no funding for this work to report.

Preliminary results were presented at the 21st annual meeting of the European Society of Pediatric Dermatology (ESPD) in Munich in May 2022, and the German Diabetes Association general meeting in Berlin in June 2022.

## Author contribution statement

TB and HO planned the study, and LH, FR, KK, JW and DJ recruited the participants and obtained the data. LH performed the dermatological investigation and was supervised by HM, JG, DJ or HO. Statistical analysis was performed by LH and TB. The primary manuscript was written by LH, revised by TB and HO, and read, edited and approved by all authors.
