# ERRATUM: Pediatric obesity and skin disease: cutaneous findings and associated quality-of-life impairments in 103 children and adolescents with obesity

**DOI:** 10.1530/EC-23-0235e

**Published:** 2024-10-14

**Authors:** Laura Hasse, Dagmar Jamiolkowski, Felix Reschke, Kerstin Kapitzke, Jantje Weiskorn, Olga Kordonouri, Torben Biester, Hagen Ott

**Affiliations:** 1 Department of Pediatric Dermatology and Allergology, Children’s Hospital Auf der Bult, Hannover, Germany; 2 Department of Pediatric Diabetology, Children’s Hospital Auf der Bult, Hannover, Germany

The authors and journal apologise for errors in the above paper, which appeared in volume 12 part 9, article ID e230235. The errors relate to the abstract (given on page 1), the main text (given on page 4) and Table 2 (given on page 4), where preliminary data had inadvertently been retained for the final published version.

**Table 2:** Laboratory results presented as median (interquartile range).

Parameter	Whole study population	<10 years	10–14 years	>14 years
LDL (mg/dL)	96.5 (18–190)	105 (49–183)	97 (18–190)	95 (66–151)
HDL (mg/dL)	49 (30–111)	50 (36–79)	52 (30–111)	48 (33–77)
Total cholesterol (mg/dL)	169 (81–265)	174 (112–265)	169 (81–265)	164 (115–216)
Triglycerides (mg/dL)	79.5 (17–379)	88 (29–200)	99 (17–379)	104 (79–241)
GOT (U/L)	26 (10–118)	28 (11–43)	29 (11–118)	22 (10–40)
GPT (U/L)	38.5 (16–250)	40 (20–98)	50 (16–250)	39 (20–65)
GGT (U/L)	18 (6–58)	17 (6–39)	20 (10–58)	22 (10–48)
Fasting glucose (mg/dL)	88 (71–318)	85 (71–101)	92 (78–264)	197 (76–318)
Urea (mg/dL)	25 (11–46)	25 (11–36)	26 (13–46)	26 (15–43)
Creatinine (mg/dL)	0.5 (0.3–1.0)	0.4 (0.3–0.5)	0.6 (0.3–0.9)	0.6 (0.4–1.0)
Uric acid (mg/dL)	5.0 (1.9–8.8)	4.3 (2.2–5.9)	5 (1.0–8.8)	5.5 (2.9–8.3)
TSH (µU/mL)	2.3 (0.8–8.0)	2.2 (0.8–4.4)	2.5 (0.9–8.0)	0.8 (4.8–2.0)
HbA1c (%)	5.5 (4.1–11.7)	5.4 (4.5–5.9)	5.5 (4.1–7.0)	4.9 (4.9–11.7)
Fasting Insulin (µU/mL)	15.0 (1.0–85.6)	16.8 (1.0–85.6)	21.1 (2.8–81.5)	23.1 (2.7–85.2)
HOMA	3.3 (0.0–14.2)	3.6 (0–21.3)	4.8 (0.6–19.5)	5.5 (21.9–24.2)

The original article abstract stated:

“Results: A total of 103 children and adolescents (age 11.6 ± 2.5 years, 41% female, 25% prepubertal, BMI SDS 2.6 ± 0.5, homeostatic model assessment (HOMA) score 3.3 ± 4.2; mean ± s.d.) were recruited in a 12-month study period. Skin affections were linearly associated with increasing BMI and higher age. The most common skin findings were (%) striae distensae (71.0), keratosis pilaris (64.7), acanthosis nigricans (45.0), acne vulgaris (39.2), acrochordons (25.5) and plantar hyperkeratosis (17.6). The HOMA score was associated with acanthosis nigricans (*P* = 0.047), keratosis pilaris (*P* = 0.019) and acne vulgaris (*P* < 0.001). The general mean QoL(QoL) score, as assessed by the WHO-5, was 70 out of 100. A total of 38.9% of participants reported impaired dermatological QoL.”

This should have stated:

“Results: A total of 103 children and adolescents (age 11.6 ± 2.5 years, 41% female, 25% prepubertal, mean BMI SDS 2.6 ± 0.5, median (IQR) HOMA score 3.3 (0; 14.2)) were recruited in a 12-month study period. Skin affections were linearly associated with increasing BMI and higher age. The most common skin findings were (%) striae distensae (63.1), keratosis pilaris (63.1), acanthosis nigricans (44.7), acne vulgaris (40.8), acrochordons (25.2) and plantar hyperkeratosis (17.5). The HOMA score was associated with acanthosis nigricans (*P* = 0.047), keratosis pilaris (*P* = 0.019) and acne vulgaris (*P* < 0.001). The general mean QoL(QoL) score, as assessed by the WHO-5, was 70 out of 100. A total of 39.9% of participants reported impaired dermatological QoL.”

The full corrected abstract is given below:

**Abstract**

Objective: Little is known about specific cutaneous findings in children and adolescents with overweight and obesity. This study assessed the association of skin signs with pivotal auxological and endocrinological parameters and their influence on the quality of life (QoL) of young people with obesity.

Study design: All patients initially recruited for a tertiary hospital's weight control program were offered participation in this interdisciplinary, single-center, cross-sectional study. All participants underwent a detailed dermatological examination, anthropometric measurements and laboratory examinations. QoL was assessed with validated questionnaires.

Results: A total of 103 children and adolescents (age 11.6 ± 2.5 years, 41% female, 25% prepubertal, mean BMI SDS 2.6 ± 0.5, median (IQR) HOMA score 3.3 (0; 14.2)) were recruited in a 12-month study period. Skin affections were linearly associated with increasing BMI and higher age. The most common skin findings were (%) striae distensae (63.1), keratosis pilaris (63.1), acanthosis nigricans (44.7), acne vulgaris (40.8), acrochordons (25.2) and plantar hyperkeratosis (17.5). The HOMA score was associated with acanthosis nigricans (*P* = 0.047), keratosis pilaris (*P* = 0.019) and acne vulgaris (*P* < 0.001). The general mean QoL(QoL) score, as assessed by the WHO-5, was 70 out of 100. A total of 39.9% of participants reported impaired dermatological QoL.

Conclusions: This study shows the high prevalence of skin lesions in children and adolescents with obesity. The association between skin lesions and the HOMA score indicates that skin manifestations are a marker of insulin resistance. To prevent secondary diseases and improve QoL, thorough skin examinations and interdisciplinary cooperation are necessary.

The original article text on page 4, ‘Skin changes’ section, stated:

**“Skin changes**

Of all participants, 88% had skin phototypes 1–3 …”

This should have stated:

**“Skin changes**

Of all participants, 86.2% had skin phototypes 1–3...”

The full corrected text is given below:

**Skin changes**

Of all participants, 86.2% had skin phototypes 1–3, which represent the most common phototypes in German minors, whereas only 9% and 4% had phototype 4 or 5, respectively (Fig. 1). Upon inclusion, 13 participants (12.6%) were undergoing dermatological treatment for atopic dermatitis (n = 4, 3.8%) and other skin diseases, e.g.,psoriasis (n = 1, 1.0%) or suspected hair loss (n = 1, 1.0%).

The original Table 2 gave a value for Whole study population HOMA as 4.6 (0.0–14.2).

This should have stated Whole study population HOMA as 3.3 (0.0–14.2).

The full corrected Table 2 is given in full below:

